# Supplementary material for: Functional Characterization of a (E)-β-Ocimene Synthase Gene Contributing to the Defense against Spodoptera litura
Source: Int J Mol Sci. 2023 Apr 13;24(8):7182. doi: 10.3390/ijms24087182 (PMC10139113; doi:10.3390/ijms24087182)
Supplement: Supplementary file 1 [file ijms-24-07182-s001.zip › ijms-2327676-supplementary.pdf]

Functional Characterization of a (*E*)-β-Ocimene Synthase Gene Contributing to the Defense against *Spodoptera litura*

Taotao Han [1], Yan Shao [1], Ruifang Gao [2], Jinshan Gao [3], Yu Jiang [1,3], Yue Yang [1,3], Yanan Wang [1], Siqi Yang [1], Xiang Gao [1], Li Wang [1,*] and Yueqing Li [1,*]

[1] Key Laboratory of Molecular Epigenetics of MOE, Northeast Normal University, Changchun 130024, China

[2] College of Plant Science, Jilin University, Changchun 130024, China

[3] Key Laboratory of Soybean Molecular Design Breeding, Northeast Institute of Geography and Agroecology, Chinese Academy of Sciences,
Changchun 130102, China

* Corresponding authors: wangli@nenu.edu.cn (L.W.);
liyq339@nenu.edu.cn (Y.L.); Tel.: +86-431-85099360 (Y.L.)

A

(E)-β-Ocimene concentration (μg)	Peak area
0.01	22915904
0.025	34881302
0.05	111161164
0.1	149614918
0.25	463456366
0.5	1045000372

B

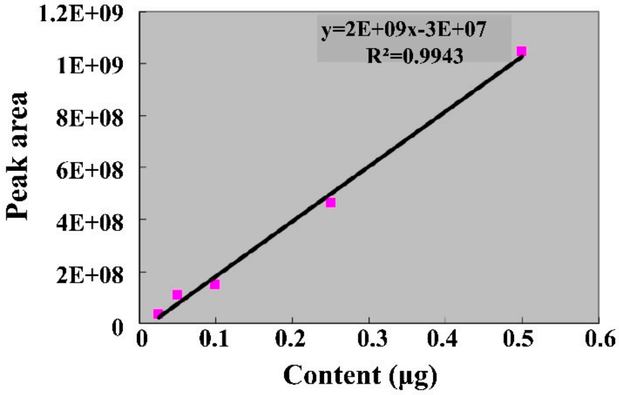

Figure S1. Standard curve of (*E*)-β-ocimene.

(A) (*E*)-β-ocimene was diluted with methanol solution to the following concentrations: 0.01, 0.025, 0.05, 0.1, 0.25 and 0.5 μg μl^{-1}. 1 μl of ocimene with different concentrations was analyzed to calculate the peak area. Peak area represented mean data from at least three replicates. **(B)** The standard curve calculated with (*E*)-β-ocimene concentrations and corresponding peak areas.

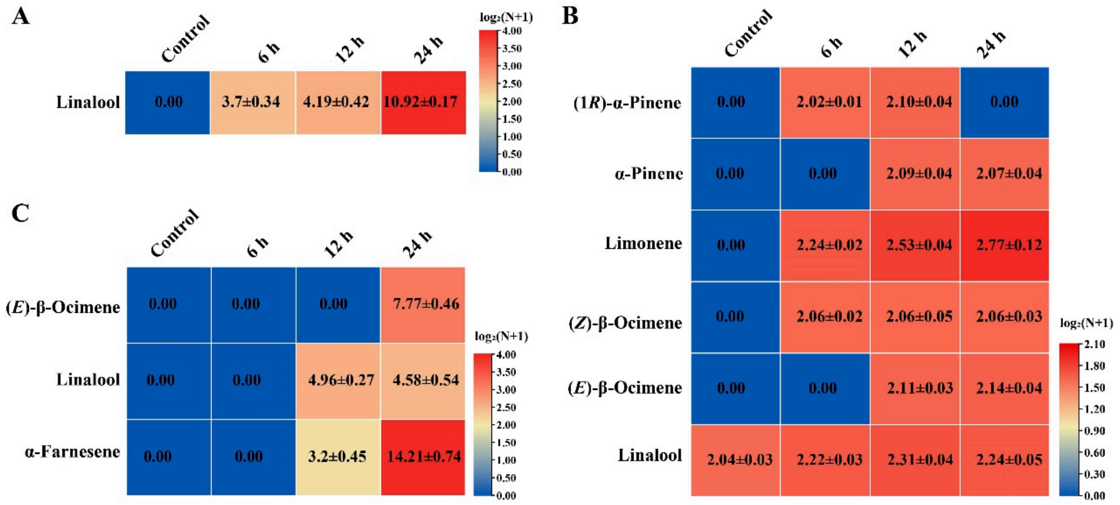

Figure S2. Relative terpene contents released from soybeans with MeJA, mechanical wounding and *S. litura* treatments.

Volatile terpene contents released from MeJA treated soybeans **(A)**, Mechanical wounding **(B)**, and *Spodoptera litura* **(C)**. Data represented mean ± SD of three replicates relative to (*E*)-β-ocimene. The data were normalized by $\log_2$ and illustrated by TBtools with HeatMap Illustrator.

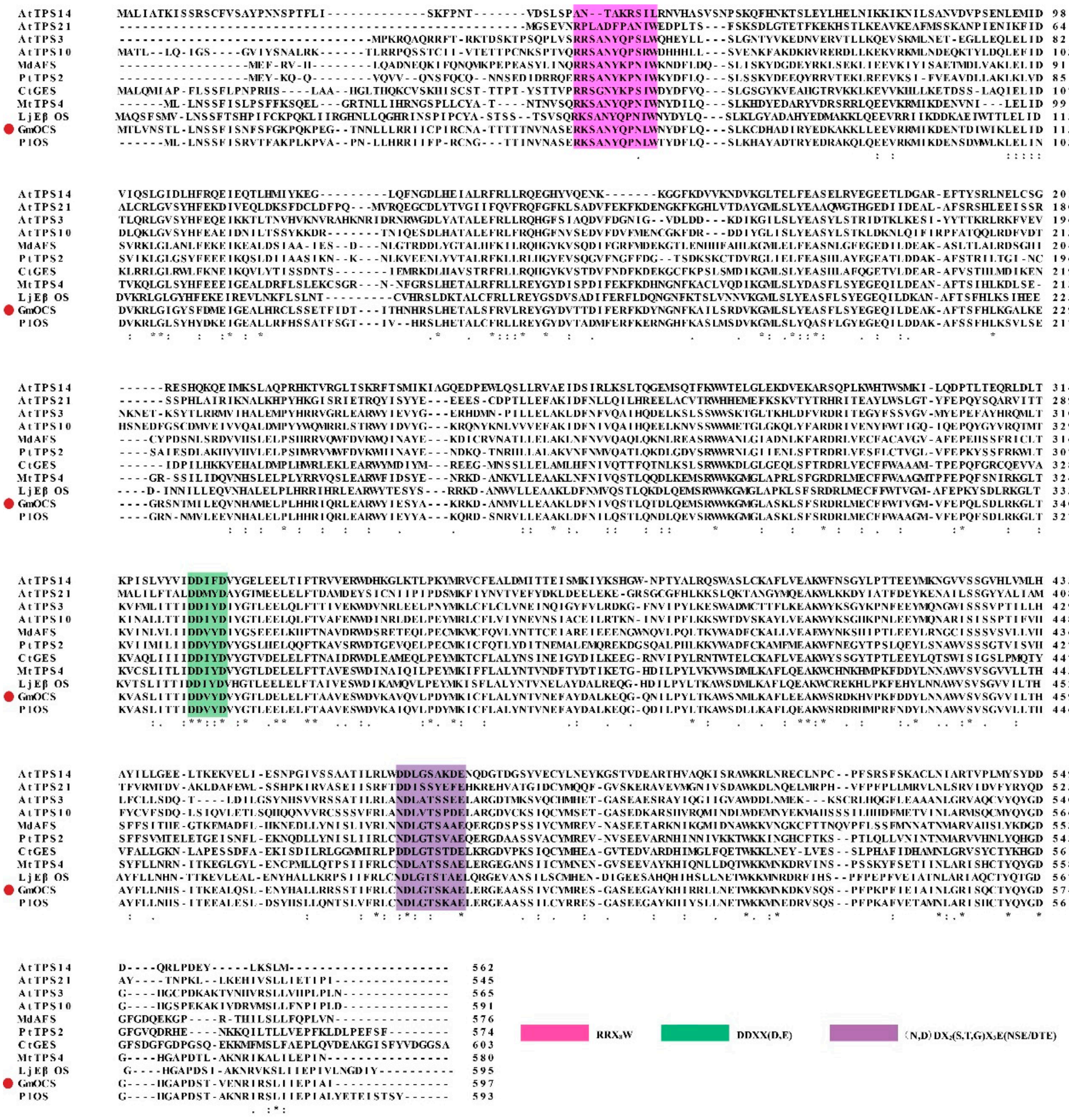

Figure S3. Multiple sequence alignment of GmOCS.

The conserved domains RR(X)8W, DDXX(D,E), and (N,D)DXX(S,T,G) XXXE (NSE/DTE) were highlighted with colored backgrounds. Numbers represented positions of the last residue in each line. *, identical amino acids, or **, similar amino acids.

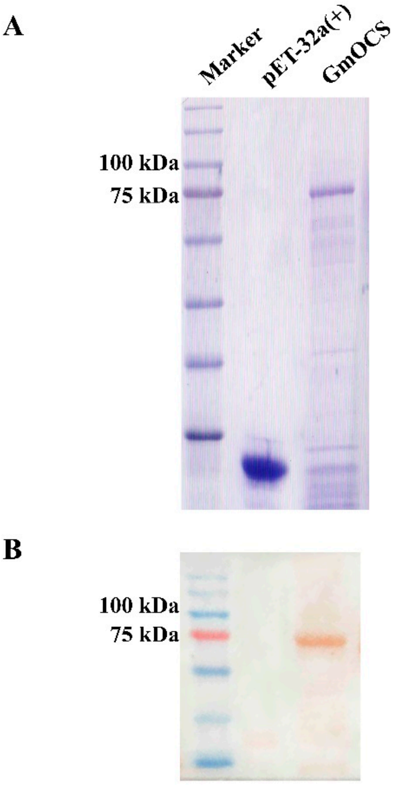

Figure S4. **SDS-PAGE and western blotting analysis of purified GmOCS.**

(A) SDS-PAGE analysis of recombinant GmOCS purified from *E. coli* BL21 (DE3) expressing pET32a. **(B)** Western blotting analysis of recombinant GmOCS purified from *E. coli* BL21 (DE3) expressing pET32a.

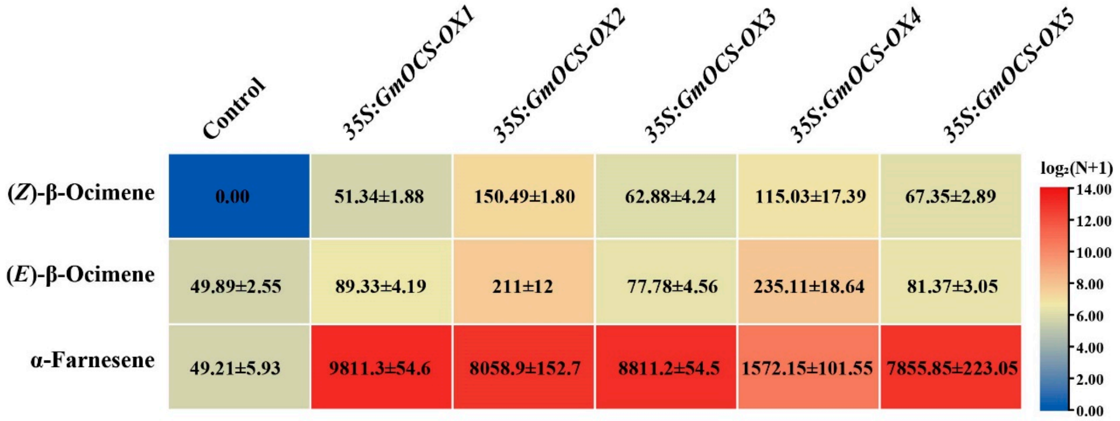

Figure S5. **Relative terpene contents released from transgenic soybeans.**

Data represented mean ± SD of three replicates relative to (*E*)-β-ocimene. The data were normalized by $\log_2$ and illustrated by TBtools with HeatMap Illustrator.

Table S1. Primers used in the study

		Forward (5'-3')	Reverse (5'-3')
**RT-PCR**	*GmOCS*	TCCTCTTTCATTTCAAATT	GGTGGTTGTTGCGTTGCATC
	GmTPS4	AAACGTGCATTTTTTCTTTC	GCGCTAGATCTTTCTTCTT
	GmTPS5	CCCTATCTTTGCTTCAGTGA	GATATTTTGGTTCAAGGCAA
	GmTPS6	ATGAAGGAGCACCCACATGT	GGATTTGCATCTGTCATGAC
	GmTPS8	CAAATGACACAACACAATGG	ATTTGTACATGCTTAACGT
	GmTPS9	CCTAGACAGCAGTTCCATGT	TTGTTGCCAAGCAGTAATAC
	GmTPS10	ACAACACAGTCTACACTTG	GCGTTGGACTGAGTCAATA
	GmTPS11	AATTAAGCCATTTCCTTGC	TACTACTGACTTCTATTTG
	GmTPS12	CCCTCACACTTCACTCGGA	TGGTTCCGTCCCGGCGTGTA
	GmTPS13	GGAAACTTCAATGAAAGGCT	CTCGAGATAAGGGCTTAAT
	GmTPS14	TTGATCTTCGGACAAACAA	GAGTGACATCCCAATCTCC
	GmTPS15	GTATATGTCTCATCTACAT	ACACCCATAGGAAGCATTC
	GmTPS16	ATGCACGTGTAGCAGAAG	TTAATGTTCATTGATGCG
	GmTPS18	CATTACCGTAGCAATCCAGG	TTCGATAACAAATTGCAGGC
	GmTPS19	TGGCACGCATGTCTATGGT	CTACTGGTGTAGTTCAAGG
	GmTPS20	CAAGAGTGGGAATTTCAAG	CAGATGAGCCCTTGAGTAG
	GmTPS21	TGGTAAAGATGTTGTGGAGC	TGTCGGTTGATGTTGATGGA
	GmTPS23	AACATCTCTTGCCAATCTCAAAC	CCAGAGGTTTGGTTGGTAAT
	GmACTIN	CTTCCCTCAGCACCTTCCAA	GGTCCAGCTTTCACACTCCAT
	β-tubulin	GGAAGGCTTTCTTGCATTGGTA	AGTGGCATCCTGGTACTGC
**Full-length cDNA sequence cloning**	*GmOCS*	ATGACCCTTGTCAATTCCACG	CTAGATGGCAATGGGTTCAAT
**Generation of constructs used in Heterologous expression in *Escherichia coli***	*GmOCS*	TGGCTGATATCGGATCCATGACCCTTGTCAATTCCACG	CGACGGAGCTCGAATTCCTAGATGGCAATGGGTTCAA
**Generation of constructs used in subcellular localization**	*GmOCS*	CTGATTACGCTCATATGATGACCCTTGTCAATTC	TTGCTCACCATATCGATGATGGCAATGGGTT
**Generation of constructs used in Heterologous expression in tobacco**	*GmOCS*	GTATATTCTGCCCAAATTCGCGAATGACCCTTGTCAAT TCCAC	ATGAAACCAGAGTTAAAGGCCTCGAGTTACTAGATG GCAATGGGTTCA
**Generation of constructs used in soybean**	*GmOCS*	ACACGGGGGACCATATGATGACCCTTGTCAATTCCAC	GATCGGGGAAATTCGAGCTCCTAGATGGCAATGGGTT

Table S2. Information of *GmTPS*s presented in this study

Name	Gene ID	TPS Subfamily	Sequence length(bp)	Function Prediction	Top BLAST match	Homology(%)
GmTPS1	Glyma.03G154400	TPS-c	2433	terpene synthase	TKY62444.1 [*Spatholobus suberectus*]	82.1
GmTPS2	Glyma.03G154700	TPS-c	2448	terpene synthase	TKY62442.1 [*Spatholobus suberectus*]	90.24
GmOCS	Glyma.06G302200	TPS-b	1794	terpene synthase	ABY65110.1 [*Phaseolus lunatus*]	81.14
GmTPS4	Glyma.07G187600	TPS-g	1722	terpene synthase	XP_027920566.1 [*Vigna unguiculata*]	76.96
GmTPS5	Glyma.07G187700	TPS-g	1767	terpene synthase	XP_013457143.1 [*Medicago truncatula*]	66.78
GmTPS6	Glyma.08G061600	TPS-g	1161	terpene synthase	XP_020238808.1 [*Cajanus cajan*]	63.68
GmTPS7	Glyma.08G163900	TPS-e/f	2505	terpene synthase	XP_020237612.1 [*Cajanus cajan*]	89.06
GmTPS8	Glyma.09G122500	TPS-b	1809	terpene synthase	XP_020210249.1 [*Cajanus cajan*]	71.97
GmTPS9	Glyma.12G102000	TPS-b	1809	terpene synthase	TKY66941.1 [*Spatholobus suberectus*]	79.08
GmTPS10	Glyma.12G138100	TPS-a	1593	terpene synthase	XP_007133006.1 [*Phaseolus vulgaris*]	71.59
GmTPS11	Glyma.12G138600	TPS-a	1662	terpene synthase	XP_020206641.1 [*Cajanus cajan*]	79.51
GmTPS12	Glyma.12G138800	TPS-a	1503	terpene synthase	XP_020207457.1 [*Cajanus cajan*]	67.99
GmTPS13	Glyma.12G140600	TPS-a	1683	terpene synthase	XP_020206642.1 [*Cajanus cajan*]	71.96
GmTPS14	Glyma.12G197400	TPS-g	1707	terpene synthase	XP_029130991.1 [*Cajanus cajan*]	81.34
GmTPS15	Glyma.12G197500	TPS-g	1782	terpene synthase	XP_027928101.1 [*Vigna unguiculata*]	75.34
GmTPS16	Glyma.12G216200	TPS-a	1695	terpene synthase	XP_020239095.1 [*Cajanus cajan*]	76.06
GmTPS17	Glyma.13G183600	TPS-e/f	2496	terpene synthase	XP_047164411.1 [*Vigna umbellata*]	73.95
GmTPS18	Glyma.13G250400	TPS-g	1605	terpene synthase	QCD76654.1 [*Vigna unguiculata*]	76.02
GmTPS19	Glyma.13G285200	TPS-a	1698	terpene synthase	XP_029126124.1 [*Cajanus cajan*]	72.55
GmTPS20	Glyma.13G304500	TPS-g	1740	terpene synthase	XP_020237811.1 [*Cajanus cajan*]	74.08

GmTPS21	Glyma.13G321100	TPS-b	1707	terpene synthase	XP_020237603.1 [*Cajanus cajan*]	
GmTPS22	Glyma.19G157000	TPS-c	2454	terpene synthase	XP_007162437.1 [*Phaseolus vulgaris*]	86.65
GmTPS23	Glyma.20G074400	TPS-b	1821	terpene synthase	XP_020210249.1 [*Cajanus cajan*]	75.7

Table S3. Enzymatic products catalyzed by GmOCS protein from *Soybean*

NO.	Compound	RI	GPP	NPP	(*E*,*E*)-FPP	(*Z*,*Z*)-FPP
	Monoterpenes					
1	(*Z*)-β-Ocimene	968	3.47±0.31	/	/	/
2	(*E*)-β-Ocimene	976	94.27±1.16	/	/	/
3	Linalool	1082	2.26±0.53	/	/	/

RI, retention index; GPP, geranyl diphosphate; NPP, nerolidy diphosphate; (*E*,*E*)-FPP, (*Z*,*Z*)-FPP, Farnesyl diphosphate; /, Not detected.

Data, mean ± SD, were the percentage of different enzymatic products.

Dataset S1. Promoter sequences of *GmOCS* and *GmTPS21*

>*GmOCS* promoter

AAATCTTAAAATAGACTCAAATAAGATTATTTTCAATAAAAAATATCAATGATTT
GGATAAAAAAAATTAATTTATCTATATAATACATTTTTATTACATTAATTCTTAATT
TTCTAATAGTATTTTTAGTCAGCATTGTACCTTGCAAGGGTCTTGATAACGGTTA
AATATGAGTTATTTTTTATAATAAAAATATATTGAAAATATCTTTAAAAAGTCAAA
AGTTAAGGCCTCTTATGACTATGAGAGGCTAAACCCCTTTTTAAAAATATCTTTA
AATAGTATTTTTAGACTCAAATAAGATTATTACATCAGTCTTTAAACCCATTTTC
AAGTGTAAGGACCCTTATGACTATGAGAGGCTAAATCTTTAGCCCTTAGTTAGG
GTCTGTAAGGCCTAAAAAGTCAAAAGGTGAATTGTACATTTCATATTTATCGAT
GCAAACATGTGTTTTCTTTCCTATTATCCTTTCTTATTTTAATTTCATGTATCATTC
ATCCTTGCATCATCTTTAGGGGTTGAGTGCTCAACAGAGGGTAATCCTTAATAG
AAATACAAGGAAGGTCTTACATGCATCAGTTTTAGGAATTAGTCGCTCGATAGA
GGATAATTTCTAATAGAACTAAAAGAAGGGGTATCTTAATAAAATCATTGCTAG
ACATAGAGTAATTGTATTATGCCCATGTATCAAAGCAAACATCTAGAATTAGAAC
TTCATGCATTTTATGTATGTATTGAGTCTTTGCAAAGACACTTGGAAGATAGATA
AGTAAGATAGATTTGTCATCGTGAGACACGCAAGGTCCAAACATTATCACATTT
TGAATCTATCTTTCTTTATCTTCGTCTTAATATTTTATTTTTCCTGTCTTTTACTTT
TGTTTTCTTATCTTTTTTTATTTTTATCATCTTTTAATTTAAATCTTTTATCTTTTCTA
TCTTTAAATCTTTATCTTATCTCCTATTTTTCTTTATCTATTATTTTATTTCCTTATC
TCTTACTTGTAAATTGAGTTTGCATCAATCAATCTAAATACAACCAAAGTCCCTA
TGGATTTGACACTCGGACTTCCGAGTACTTTACTACTTGTGACAAATTGGTGCA
CTTGCCAACGAGTTAACAAGTTTTTGACACCGTTGCTGGGGACTTTGTTATTCT
TACTTAGTTGTTGCATATTCCAATTTTAAAGCAATCAATTTTCATCTTTTCACTTT
TTATTTTATTTTTCAAAAAAAAAAAATTTACTTGTGAGTCTATGCTTGCAGGGTG
AAACCTCAGAAGAACTTGATCCGTGGAAGACATTTTAAAGATGGAAGATCTTT
TTAAGAGGTGAACTCAGTATCTTGACAGCTCACGATCAAGGGTCAGTCACCCA
CCACCGGTAAATTGTGATTTTTGTGAAGGAGAGCATTGCAATGACAACTGTCAT
CTCTACTCCATGAAAAACTCTTGGTGGGGATAAGAGTTACACCTTTACAATCAA
TATGAAGAAAAAAGAACTCCTAATCTTGAGGGTGTGTTCACAGAATTCATGGC
ATACCATGCTAGCTCTACAACCAATCAAAACTTTATGTAAAACCAAGAAATTCA
GGTTGGCAAGAGCTACTCCATGGAAAATTATTGGTGGGAGTAAAAGTTATAAC
CCTACAATCAATATGAAGAAGAAAGAAGTTCTGATTTGGATAATTTGTTGATGC
AATTCAAGGAAACAACTGAATCTACTCAACGAGCATTTAAAAGTGTAGAAATT
CAAGTTGGTAAGCTAGCAAAAGAAGTGGCTAAATTTGTGGCCACAAGGGAAG
AAAACTTTGTAAAGGTAATCATGTTTTTTGTAAAACCCCTAAGCCTGCTTTAGT
TACCTCCTACCTTGTTTTAGGGATTAAGATTGCATAAGGATTTTACTCGTGATAT
GTCCCTAATTTGGGGGAGGGTTAAGGTAAAAATTGTCTCTGGAAGGTAAAACA
ACATACAATAAAGCACCCTAAAAAAAGCTTGTAAGCCCAAAGTTTCTTTCCTA
AAAAAAAAGGAGATAAATCCTAAAAAAAAGAAGAAAGAAAAACAAAAGAGA
AGAATAAGGGCAGAAAATAAATAGGTGTCATAAGTGTTGTTAGAACAATAAAC
TGAGGCTGAAAAACAAATAAGCCTAGGCTGAATAAGTGGAAGTTTTTATGGGA

TAAATGCTCTCTTATAACCCTAATTTTTGAAATCCCAAAAAAACTATAATTTCTT
TTATTAGCCAGGTCATGTTACAAGCCAATAAAAGTCCTTAGTGATCCACCAAGT
GTAAGCATGATAACTTTAACTGAGATGAAGTGCAAAAATTGAGAACATTAATTG
CATGCCGTAGAATTTTAAACATTCACCCAAGACACTTGTGCGCAAAGAGAAAC
ACTAAAACCTTGTGAGGAAAAGTGAGGCAAGCCAACCTGATTGATTTCTACTA
CTAACCAATTCTATCTCAATGTTTGTTTCTACTTCAATTGCAAGATCATGGTAAA
TGCAAGAAAGTCCAACTAAGGGAATTTGAAAAATGGCAGCTATTGAAATAAAA
ATATATTGAAAATATCTTTAAAAATATTTATTTAGTATTTATTTTTTACTTAAATATT
AGGAATTGATATTTTTCTTATTTATGACTTGTAGATATGAAAAAGAGGGATTAAA
AGCAAAAAAATCTAGAAAATATCCAAAATATAAATAAGGATTATTTTTTGACAT
CAAGCCCAAATTCACTCCAGCAACTATAGAAAGGGAGTCAAGCCCAAGTCCA
CTCCAGTAGCTAAAAAGGGAGTCAAAAGGAGAAAAGACACCACCCCGAAGA
CTCATAGCTCTCTAATGAATACATCCTAAGCGTGAGTGTCTCTAGTAAGGGAAA
TCTTTCTTTTTCCATCCCTTCTCTTTCATCAGTTTCTAAATCTCTTTTCAAGTGTA
AGGTCCCTTATGGCTATGAAAGATTAAATCCTTAGTTAGGGTCTGACAGACCTA
AAAAGTTAAAAAATGTATTGTACACTTCATATTTATCGATCCAAACAAGTGTTTT
TTTTTCCTATTATCCTTTCTTATTTTAATTTCATGTATCTTTCATCATTGCATCATCT
GTAGGAGTTAGGTGCTCGACAGAGGATAATCCTTAATAAAAATACAAGGAAGG
TCTTACCTGCATCAATTTTAGGGATTAGTCGCTTGACAAAGGATAATTTCTAATA
GAACTAAAAGGAAGGGGTATCTTAATAAAATTATATCTTCTTTTACTTTTCTTAT
CTTATCTTTTTTTATCTTCTATTTTTTATCTTTATCATCTTTTATTTTAAATCTTTTAT
CTTTTCTATCTTCTATTTTTATCTTTAAATCTTTTTATCTTATCTCCTATCTTTCTTT
ATCTATTATTTTATTTATTTATCTCTTACTTGTAAATTCAGTTTACATCAATCTAAA
TACAACAAATATTGTGACAAATTGATGTACTTTCCATCGATTTATCAGATCTTTC
CTAGGACCATTCTTTATGCCATACTTTTTTGTCTTATGTTTGAAGAGGTCTATCTT
AGGATAATTTCTTATGTGATATTTTTTTGTCTTATGTTTGGAAACTTCAAATTCAT
GATCTTATCACTTGAAACATCTCACATCATATCTTATATCGGTTTCCAAAGGTGC
TTCTTCTGTATTCAACTTTTTTTTTAAGTTGTAAAAAAATTTAATTAAATACTACT
AAATTTTGTTGACCTCCTTAAAAAAACCTTAATATAATCCTAATTTTATTTTAAA
AATAAAATATTTTATTAACAAAAATAATAGCATGACCACAAGGAGTGACCAAAC
TAACACCCAAATACACCACAGACCAAAAAGAAAATAACATGACCACAAGAGT
GACCAAACTAGCACACAAATTCTTTGAACTAACAGTAATCAAATGTTGCAAGC
AATATTAATTTTTAATTTAATGGATTTAGAGACATTGATAATCTTTAAAAAAAAAT
ATGCACCACAAAATACTCGTTGAATATCTTCCCGGCAGAGATCCTACTTCCAAA
CACCTGTCCTACTTCAATATAGTACATTTAGCATGCGCTGCATACACAAAGGCC
ACATATAAAACGTGAACACAAATAATTCATAGCTATAGACCCTGCATGGTGTGG
CTATATATAAACGTGACCACCCGGAACATCAAATGCAAGTAGCTAGCACACAGT
ACTACTGTTAAACATTCTCAGTTGCAAGCATAAGCCAACCTAGGATCGATAAAA
ATG

>*GmTPS21* promoter

TTAGATTTTTTTATCTTTATCATTTATATTTCCTTTGTCAAATTCAGTAATTTAAC
CAGCTACTTGGAACTGCTGCAGGTACCTACTGATCAGCCTTGCATAGCACTAAA
CATGGTGGGTGCAATTACACAGTCACCTGCGGTTTGAAGCTAGTTATATTAAAT
CATATAGAATAAGTAAAATTGACTACTACAAGTGATTAATAAAGAGAACCACAG
AAAAATCAGAAGGAAAGAGTTCACAATCAAACTATAACGGAATCAAACAAAG
AATAGTGAGAACTTCAGGGAAGATGCCATGAAATGTTAACATATGTTAACAGAT
TTTAACATGCATGTACAATATCGATCATTCTATACTTATGGGATTACTATGTGTTT
TTTCTTCCTTATGAAGTTTCGCCATTTCTTTACTTTCGGGGAGTGGATTGAAAA
CTTAAAATGCAGTGGCAACACAAATTGGTGAAGTTATAATAATAATAATAATATT
ATTATTATTACACATTATATAATTTTATTAAAGTTTCAATCATTATTTTTTAATTAAC
TGTCACAAATTTGCAATCATAAGCTCTCTCTATATATATTACCTTTTTTTTTTAAG
AAGTCAAATTCAATACATTCTCTCTTTGCAAGCATGGGCACCTAACTAAATTCT
TTGCACGCATTAAAAGTTTTTTTTATTAACAAATGTTATTTATTACTTATTAGTTT
TTGTTAATAGAAAAGATTCAAATTCACTATCTTTCTTTTCTTACTTCAACTGTAC
ACATTAAAAGTTAAATTTCAAACACATGAAATATACCACCAAACCAATGTGGAT
CAATAAGTGTAGGCTTATTTAAGCTTAATCAGTTTAGAATTTAACTATTGCATAT
GTAATAATTTTATTTAATTTAAACAAAATTATGTTCCATGGAAGTTGGATTCATGT
GATTCATTTGGCAAGACAGCGACACTATCACCAATATTTGCAATCCAAGTTATG
GAAAAATGCGTGACAACTTACATATTGTCAAGGGCCGATTTAACCCTTTTGTTT
GATTCAGCAACATTAATGGTTGGTGGATAGAAAAGGGGAGGGTAATTGGTCTG
AAAATACTTACTCCTTGAGTAAAATATAAGAAAGAAAAGCATGACACAATAATA
AAAAAAATTACTTAATCACATCAATTTTAATTAAAAAAATTTAATTTACTTTTTAT
TGGAATTTGATATTAGAAAAAGTTATTGAAATTAACACATCAATGAGATAAAAT
TAATTTAAATTTTCTTATATTTCATACAAAATATATATTTTTTATATTTAAACCAAA
AAATGTATATTAATTGATATTAAAAAACGCACGTTTTCAGGAAGGAAATGAAAA
GCTTAAACGCCGTTTATTTAGTTAGAGTTTTTATGGGACATTATTTAGTTAGAGT
TTAAGTTTTATATATGCACTTTAGTCTGTCAATGTAAAAATATTATTACGTCTTTA
ACTATTTATAAAAAATAGTTTAAAATCAATAATTTACTATTATTTTATTAAGTTTTA
TTTCGTATAAGCCATCAGTTAGTATTAATTAATTGCATGCTTCATTATTTTATAACT
TACAATTAGCATTCAATAAAATTATTAAAATTATTTTTTAACTTTTTTTTTTACTTT
TTTATTACTCACAATTTCTCACATAAAATATTCTAAAATAATTAAAGATATGAAAT
GTATGAATAGTATGATATAAAAAAGACTTAAATGTAAAAAAGACTTAAATGTAA
TTTTTTTCTTTCCATTTTTAAAATTTGTAATTTAATCTCTGTATTTTAAAATCATGC
TTGATATAACTCTAATCATAATTTTTTTTTATCAAATTTAACAAATTTATCATACAT
CTACTTCTCATATTTTTTTAAATCTATAATTTTAATTTTTTTAAATTGAGACATTTA
ATTCTTTAATTTTACAAAATTTATGATTTTGATCCTTCCAAAACCAAATCAGTGA
CCAATACAAAATTGATCCTTCAAAATTAATAATATGAAATAAAATTATTTGTTTA
ATCTAGGTTACATCAACCTTAATTTCTAGACAAATCAACATCATAAATTTTTTAA
AAAATTGAGAAAGTAAATGTTTGTATTTAAAAATTAAAATTACATTTAAACCTA
AAAAAAATCTTTGGCTCACATCTTAAATCGATAGTACAAAGATGTAAAATATAA
CCCCACCTTTCATTATGTACGTTTGCATTTAGCTGGATGCCGGATTCCACATGTT
TGTAAGTATATATACAAATCTTATTTTTTTAATAGATGTCACGTAAGTACAAACTA
TGGGTTGTTGAATAATAATAGTCCAGTCCAACTCTTGAAGAGTTGAAGGCCTA

GGAGCCACAAATGTTGGTCTATGGGAGATATTTTAAAAATATAAAATAAAATTAT
CAAAAGAAAATTAATATTTGTATTATAGGTTAATAAAATTGTCCTTAAATATAAG
TTGAGCCAACCAGTTAATCCAAACCGCACAGGCCAGTTAATCCATAAAAATATA
AGTTGTCATTTAACCTATAAAATAATAAAATATAATACATAATAATGGAAATAAA
ATATTGACCTTAAAATAAACATTTGTATTACATATATAATATTAAATTTGTCCTTTG
TAATTTTACTAATTATAGAAAAATAAATAATAAAAAATAAATCGAGCCAACCAGT
TAATCCAACCCGCACAGGCCAGAAAATTTTTGGGCTGGACCAGGCGAAAATAA
ACAAAATATAGTGTACTGTGCATTAATTCCAAATAATAAATACGACAAAAGAGT
TGTGATGTGATTTAATTACAACAAATTATTTTATTTGTAATGTCATTTATAAATGT
ATGAGCCACAAAATGTTCCATCTTAATTTTCTCGAAAATATGAAGAAAAAAAA
AAGAATATGGAGGACAGCCACATGTTTCTAGATTTGTATGCGAGTATGTGGCCT
ATATAAAGGTTGTGAGCTTTAATTTGGTTAGAAGAATAACAAAATAACAAATAT
CAAAAGGTTCGCAGCTAGGCAGGAACAATG
